# Psychological Impact of COVID-19 Epidemic on Adolescents: A Large Sample Study in China

**DOI:** 10.3389/fpsyt.2021.769697

**Published:** 2021-12-06

**Authors:** Jiawei Zhou, Yini Wang, Tianyi Bu, Sijia Zhang, Haiyun Chu, Jiarui Li, Jingyun He, Yansheng Zhang, Xuan Liu, Zhengxue Qiao, Xiuxian Yang, Yanjie Yang

**Affiliations:** ^1^Department of Medical Psychology, Harbin Medical University, Harbin, China; ^2^Department of Cardiology, The Second Affiliated Hospital of Harbin Medical University, Harbin, China; ^3^Department of Medical Education Management, Harbin Medical University, Harbin, China

**Keywords:** COVID-19, adolescents, depression, anxiety, posttraumatic growth

## Abstract

**Background:** The COVID-19 pandemic is a major public health emergency. However, little is known about the psychological impact of this pandemic on adolescents. We aim to assess the prevalence and influencing factors of depression, anxiety, and posttraumatic growth (PTG) among adolescents in a large sample study.

**Methods:** This cross-sectional study collected demographic data and mental health measurements from 175,416 adolescents covering 31 provinces, centrally administered municipalities, and autonomous regions in mainland China from February 23 to March 8, 2020. The status of depression, anxiety, and PTG was assessed by the nine-item Patient Health Questionnaire, seven-item generalized anxiety disorder questionnaire, and post-traumatic growth inventory.

**Results:** The prevalence of depression, anxiety, and PTG in adolescents was 35.9, 28.0, and 45.6%, respectively. The prevalence of depression and anxiety in the slight or severe epidemic areas was similar. Regression analysis showed that female sex and older age were associated with higher levels of depression, anxiety, and lower levels of PTG. Symptoms related to COVID-19, excessive attention to epidemic information, living in urban or severe epidemic areas, and conflicts with parents during home quarantine were risk factors for depression, anxiety, and PTG. Frequent communication with peers, exercise, and receiving public welfare psychological assistance were protective factors. Poor family economic status was a significant risk factor for depression and PTG.

**Conclusion:** Our findings suggested that positive and negative psychological reactions coexist in adolescents faced with the pandemic. The factors associated with psychological problems and PTG provide strategic guidance for maintaining adolescents' mental health in China and worldwide during any pandemic such as COVID-19.

## Introduction

The novel coronavirus disease 2019 (COVID-2019), which was declared a public health emergency of international concern by the World Health Organization (WHO), swept across 210 countries and territories with over 5.2 million cases and 337,736 deaths reported as of May 24, 2020 (1). China and many other countries closed their schools and relocated their classes online in response to the COVID-19 outbreak. Consequently, in China, more than 220 million children and adolescents were confined to their homes. Under these circumstances, adolescents experienced exceptional stress and challenges.

A recent study demonstrated that uncertainty about the personal and global effects of COVID-19 impacted the psychological condition of the Chinese public (2). Adolescents could be considered as a more vulnerable group because of their inadequate understanding of the epidemic, the erroneous information caused by cognitive immaturity, and the fact that excessive attention to epidemic information may potentially exacerbate stress, depression, and anxiety. Furthermore, adolescents establishing independence begin to prefer communicating with friends to parents, which may also influence their psychology. School activities and social relationships are the main methods of coping with these problems (3). However, during this unusual period, a lack of in-person contact with peers may negatively influence adolescents' mental health. Meanwhile, adolescents inevitably had the most contact with their parents, who were highly authoritative in many Chinese families (4), which tended to trigger parent–adolescent conflicts (physical or verbal conflict) and further aggravated the detrimental effects on their mental health. Moreover, staying at home for a long period may increase the risk of establishing unhealthy behaviors, such as relatively less physical activity and long screen time (5). Thus, the interaction between psychological stress and lifestyle changes during the COVID-19 outbreak may adversely affect adolescents' physical and mental health, which can cause a vicious cycle.

Although the sudden outbreak of COVID-19 resulted in negative consequences, positive changes were also observed in adolescents as an adaptive response to this global crisis. As proposed by Tedeschi and Calhoun (1995), posttraumatic growth (PTG) occurs after a potentially traumatic event, leading to enhanced personal strength, openness to new possibilities, deeper relationships with others, greater appreciation of life, and spiritual development (6). During the pandemic, adolescents quickly adapted to the online learning style, improved their self-sufficiency skills, and were more appreciative of their health, family, friends, and life. Some young people even participated in volunteer services to support the control of COVID-19 (7). These positive changes during the COVID-19 outbreak could be considered a reflection of PTG.

The present study conducted a survey from a large adolescent cohort in mainland China, aiming to (a) describe the prevalence and distribution of depression, anxiety, and PTG among adolescents during the pandemic, to explore whether challenges and opportunities coexist for adolescents in the face of the COVID-19 outbreak; and (b) investigate the risk and protective factors of depression, anxiety, and PTG among adolescents. This study contributes to identifying high-risk groups and providing strategic guidance for adolescents' mental health in all countries affected by COVID-19.

## Materials and Methods

We conducted this online cross-sectional survey in mainland China from February 23 to March 8, 2020. Chinese adolescents aged 16–26 years old were invited to participate in the online survey through the Survey-Star platform. The electronic questionnaire was distributed through the WeChat group, and the participants were encouraged to pass it on to others. In total, 175,416 adolescents covering 31 provinces, centrally administered municipalities, and autonomous regions in mainland China (excluding Hong Kong, Macau, and Taiwan) were included in the present study during the COVID-19 outbreak.

The present study was approved by the Ethics Committee of the Harbin Medical University (HMUIRB20200002). The attributes, benefits, uses, and disadvantages of the study were explained to all participants. Each participant provided online informed and written consent to participate in the study.

### Procedures

The nine-item Patient Health Questionnaire (PHQ-9) was used to measure participants' depressive symptoms. The PHQ-9 is a self-reported questionnaire consisting of nine items. Each item of the questionnaire is scored on a four-point scale ranging from 0 (not at all) to 3 (nearly every day). The total score was obtained by summing all items. Higher scores indicate more severe depressive symptoms, and the cutoff score for depression in the present study was 4. The PHQ-9 has previously been shown to have good reliability (Cronbach's α = 0.85) and has been widely used among the Chinese population (8). In this study, Cronbach's α for all nine items was 0.906.

The seven-item Generalized Anxiety Disorder Questionnaire (GAD-7) was used to measure the anxiety symptoms of participants, which included seven items. The items are rated on a four-point scale ranging from 0 (not at all) to 3 (nearly every day). The seven items were summed to generate a total score ranging from 0 to 21. Higher scores indicate more severe anxiety symptoms, and the cutoff score for anxiety in the present study was 4. The GAD-7 has previously been shown to have good reliability and has been widely used in the Chinese population. In this study, the Cronbach's α value for all seven items was 0.91 (9).

The post-traumatic Growth Inventory (PTGI) was used to measure the positive changes of the participants during the COVID-19 epidemic, which was developed by Tedeschi and Calhoun (6). The scale included 21 items rated on a six-point scale ranging from 0 (no change) to 5 (very great degree of change). The scale had five dimensions: personal strength (four items), new possibilities (five items), relating to others (seven items), appreciation of life (three items), and spiritual change (two items). The scale score was calculated using the sum of all items with high scores indicating a higher level of PTG. The cutoff score for PTG was 60 (10). The scale demonstrated good internal consistency, and the Cronbach's α value for all items was 0.958 in this study.

Sociodemographic data included the participants' gender, age, residential areas (rural/urban), situation of the COVID-19 epidemic in residence, whether cumulative confirmed cases were >500 (slight/severe), and family economic situation (poor/good). Moreover, the following further information was collected: symptoms related to COVID-19 during the past 14 days, including fever, cough, and fatigue (no/yes); attention to epidemic information per day (>5 h/3–5 h/ <3 h); conflict with parents during home quarantine, including verbal or physical conflict (no/yes); communication with peers (seldom/sometimes/frequently); exercise for 60 min per day during the epidemic (no/yes); and receiving public welfare psychological assistance during the epidemic (no/yes).

### Statistical Analysis

The Statistical Package for Social Sciences 22.0 (SPSS 22.0) was used for all statistical analyses. Continuous data were shown as mean ± standard deviation. Categorical data were presented as percentages. *T* tests or one-way analysis of variance (ANOVA) were used to examine the associations between continuous variables. Categorical data were compared using the chi-square test. Multiple linear regression analysis was used to identify the factors associated with anxiety, depression, and PTG. Statistical significance was set at *p* < 0.05, which were regarded as significant for all tests (two-sided).

## Results

In total, we collected 175,416 valid questionnaires, namely, 80,695 (46.0%) males and 94,721 (54.0%) females. The average age of the study population was 20.50 ± 1.78 years (mean ± SD), ranging from 16 to 26 years old. The mean scores for depressive symptoms, anxiety symptoms, and PTG were 4.15 ± 4.78, 3.18 ± 3.61, and 53.54 ± 21.44, respectively. The prevalence of depressive and anxiety symptoms among Chinese adolescents was found to be 35.9 and 28.0%, respectively. Notably, 45.6% of adolescents experienced PTG during the COVID-19 epidemic. Furthermore, Table 1 presents the scores for each dimension among adolescents who experience PTG. The dimensions with the highest means were spiritual changes, personal strength, relation to others, appreciation of life, and new possibilities.

**Table 1:** Post-traumatic growth scores and dimensions in the adolescents who experienced PTG (*N* = 80,025).

**Dimensions**	**Minimum**	**Mean (SD)**	**Mean item score**
Relation to others	1–35	23.80 (4.42)	3.40
New possibilities	5–25	16.54 (3.26)	3.31
Personal strength	1–20	14.18 (2.68)	3.54
Spiritual changes	0–10	7.12 (1.49)	3.56
Appreciation of life	1–15	10.15 (1.93)	3.38
Total score of PTG	60–105	71.79 (10.92)	3.42

*PTG, posttraumatic growth; SD, standard deviation*.

The basic demographic data, distribution of depressive and anxiety symptoms, and PTG are shown in Table 2. The results showed that there were significant differences in depressive symptoms related to gender, residential area, situation of the COVID-19 epidemic in residence, symptoms related to COVID-19 during the past 14 days, attention to epidemic information per day, family economic situation, conflict with parents during home quarantine, frequent communication with peers, exercise during the epidemic, and receiving public welfare psychological assistance during the epidemic. Except for residential areas, other basic demographic variables showed significant differences in adolescents' anxiety scores. Except for age, other basic demographic variables showed significant differences in adolescents' PTG scores (*p* < 0.05).

**Table 2:** Demographic data and the distribution of depressive symptoms, anxiety symptoms, and PTG.

**Variables**		***N***	**PHQ-9 score**	**T/F**	**GAD-7 score**	**T/F**	**PTGI score**	**T/F**
Gender	Male	80,695	3.68 (4.97)	−37.99*	2.84 (3.89)	−36.40*	54.99 (22.96)	26.21*
	Female	94,721	4.55 (4.58)		3.47 (3.33)		52.30 (19.96)	
Age (years)	16–20	93,872	4.14 (4.75)	−1.07	3.08 (3.53)	−12.67*	52.67 (21.24)	−0.78
	21–26	81,544	4.16 (4.82)		3.30 (3.69)		53.51 (21.67)	
Residential areas	Rural	72,547	3.96 (4.64)	−13.28*	3.16 (3.54)	−2.29	53.59 (20.70)	6.48*
	Urban	102,869	4.27 (4.87)		3.20 (3.65)		53.31 (21.91)	
Situation of COVID-19 epidemic in residence	Slight	124,673	3.98 (4.69)	−22.75*	3.07 (3.55)	−20.26*	54.15 (21.32)	18.61*
	Severe	50,743	4.56 (4.98)		3.45 (3.74)		52.05 (21.65)	
Having symptoms related to COVID-19	No	174,346	4.13 (4.76)	−23.30*	3.16 (3.58)	−30.10*	53.57 (21.42)	7.95*
	Yes	1,070	7.54 (6.95)		6.49 (5.59)		48.34 (23.52)	
The attention to epidemic information per day	>5 h	663	8.00 (6.45)	2,510.71*	11.70 (7.96)	1,170.01*	35.98 (24.11)	−1,781.16*
	3–5 h	7,218	4.56 (4.55)		7.25 (6.19)		39.97 (21.37)	
	<3 h	167,535	3.10 (3.52)		3.99 (4.62)		54.19 (21.20)	
Conflict with parents during home quarantine	No	151,364	3.77 (4.46)	−85.90*	2.96 (3.41)	−65.26*	54.58 (21.10)	50.85*
	Yes	24,052	6.56 (5.89)		4.58 (4.40)		47.06 (22.41)	
Family economic situation	Poor	29,824	4.44 (4.98)	11.60*	3.32 (3.71)	7.23*	51.31 (21.69)	−19.78*
	Good	145,592	4.09 (4.74)		3.15 (3.59)		54.00 (21.36)	
Communication with peers	Seldom	13,862	6.80 (6.74)	3,874.60*	4.71 (5.10)	1,988.67*	41.24 (24.09)	−7,462.51*
	Sometimes	63,015	4.71 (4.82)		3.44 (3.59)		48.34 (19.91)	
	Frequently	98,539	3.42 (4.21)		2.80 (3.28)		58.60 (20.46)	
Exercise during the epidemic	No	75,421	4.43 (5.14)	21.28*	3.37 (3.91)	19.46*	49.09 (22.06)	−76.72*
	Yes	99,995	3.94 (4.48)		3.03 (3.35)		56.90 (20.32)	
Receive public welfare psychological assistance during the epidemic	No	85,044	4.54 (4.94)	33.53*	3.35 (3.68)	19.65*	52.76 (21.13)	−14.75*
	Yes	90,372	3.78 (4.60)		3.02 (3.54)		54.27 (21.70)	

PHQ-9, Nine-item Patient Health Questionnaire; GAD-7, 7-item Generalized Anxiety Disorder Questionnaire; PTGI, post-traumatic growth inventory; ^*^p < 0.05.

**Figure 1:**
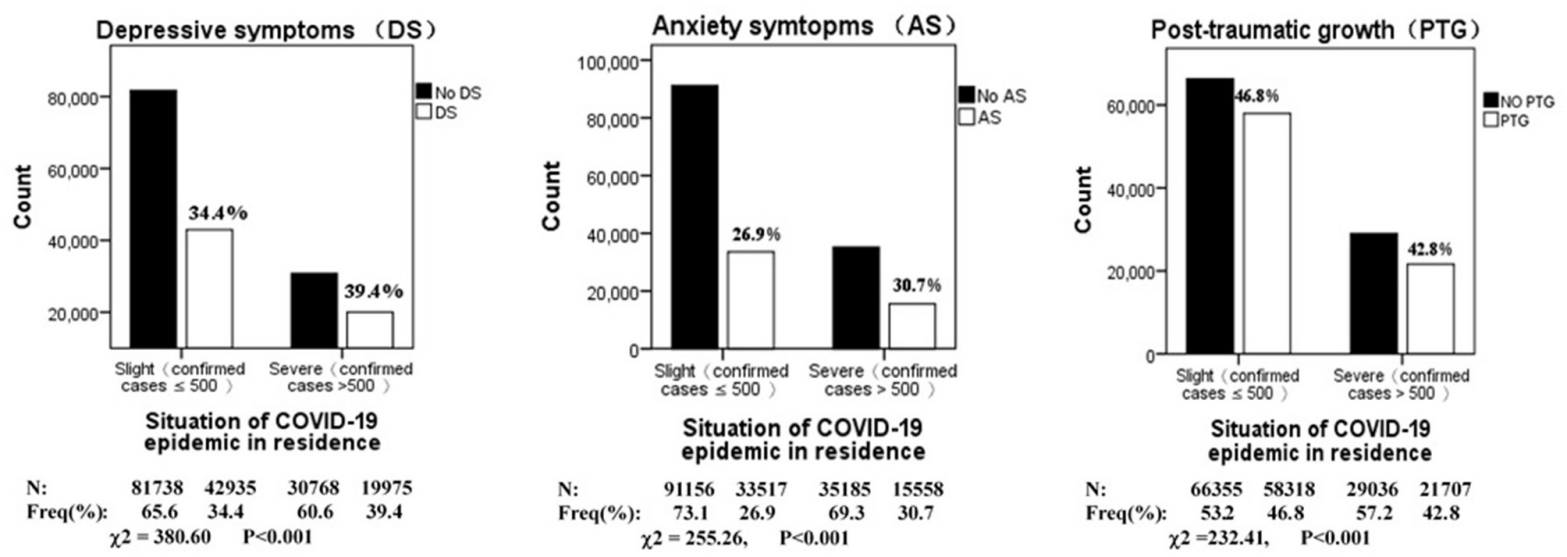
Prevalence of depression, anxiety and PTG during COVID-19 outbreak in China, stratified by situation of COVID-19 epidemic in residence (*N* = 175,416).

The prevalence of depression, anxiety, and PTG during the COVID-19 outbreak in China stratified by the COVID-19 epidemic in residence is shown in Figure 1. Although statistically significant differences were found in the prevalence of depression and anxiety according to the COVID-19 epidemic, the differences were due to the large sample size (*N* = 175,416). Figure 1 shows that the prevalence of depression was 34.4% in slight epidemic areas and 39.4% in severe areas. The prevalence of depression between the slight and severe epidemic areas was small and almost identical. Similar to depression, the prevalence of anxiety was 26.9% in slight epidemic areas and 30.7% in severe areas. Therefore, we indicated that there were no differences in the prevalence of depression and anxiety stratified by the COVID-19 epidemic in residence. Similar results were observed in case of PTG.

**Figure 2:**
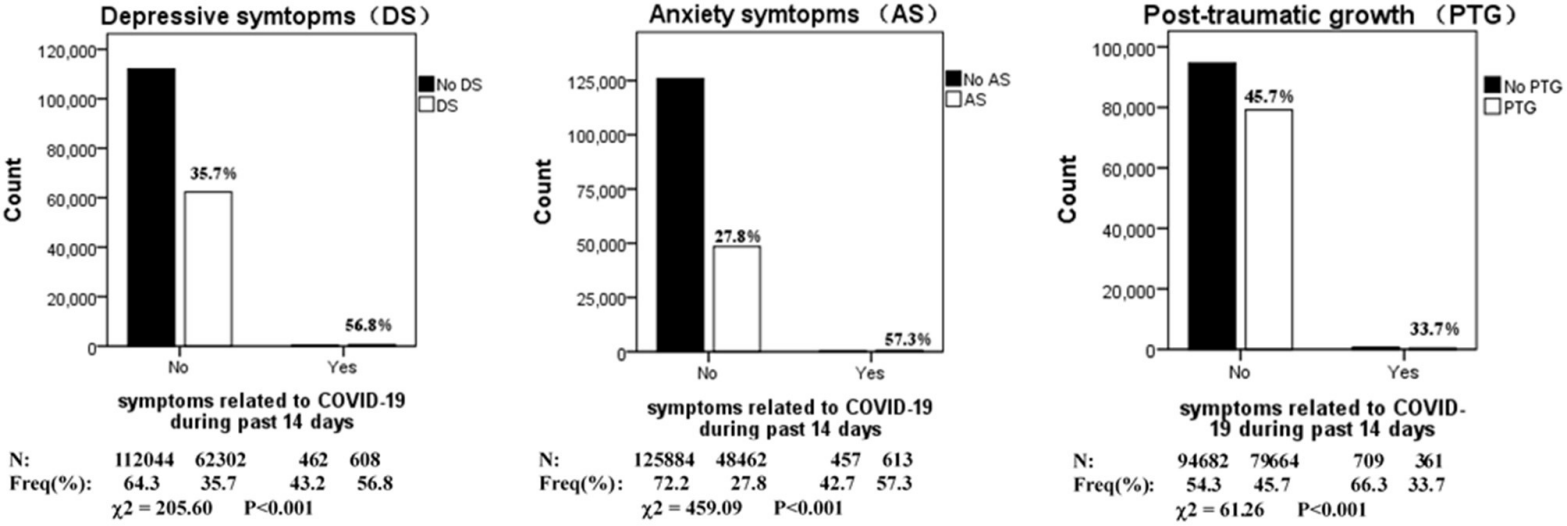
Prevalence of depression, anxiety and PTG during COVID-19 outbreak in China, stratified by symptoms related to COVID-19 during past 14 days (*N* = 175,416).

The prevalence of depression, anxiety, and PTG during the COVID-19 outbreak by symptoms related to COVID-19 during the past 14 days is shown in Figure 2. Statistically significant differences were found in the prevalence of depression, anxiety, and PTG between people who had symptoms related to COVID-19 during the past 14 days and those who did not (*p* < 0.05).

Multiple linear regression was conducted to determine the influencing factors of depressive symptoms among Chinese adolescents, and the model was highly significant (*R* = 0.32, *R*^2^ = 0.10, Adj*R*^2^ = 0.10, *p* < 0.001) (Table 3). Our results indicated that older age, female sex, living in urban areas, severe epidemic areas, symptoms related to COVID-19, poor family economic situation during the past 14 days, and having conflict with parents during home quarantine were risk factors for depression. Among the risk factors, symptoms related to COVID-19 had the strongest impact on depression. Focusing on epidemic information (<3 h/day), frequent communication with peers, exercising, and receiving public welfare psychological assistance were protective factors against depression experienced by the participants. Moreover, frequent communication with peers had the greatest positive influence on depression.

**Table 3:** Results of multiple linear regression analysis of influencing factors for depressive symptoms.

**Variables**	**B**	**SE**	**ß**	***T***	***p***
(Constant)	11.44	0.16		72.89	<0.0001
Gender	0.97	0.02	0.10	43.93	<0.0001
Age (years)	0.09	0.02	0.01	4.06	<0.0001
Residential areas	0.31	0.02	0.03	13.70	<0.0001
Situation of COVID-19 epidemic in residence	0.35	0.02	0.03	14.45	<0.0001
Having symptoms related to COVID-19	2.96	0.14	0.05	21.27	<0.0001
Family economic situation	−0.06	0.03	−0.01	−2.07	0.04
Conflict with parents during home quarantine	2.06	0.03	0.15	62.79	<0.0001
The attention to epidemic information per day	−2.06	0.05	−0.10	−42.71	<0.0001
Communication with peers	−1.24	0.02	−0.17	−70.89	<0.0001
Exercise during the epidemic	−0.45	0.02	−0.05	−20.13	<0.0001
Receive public welfare psychological assistance during the epidemic	−0.59	0.02	−0.06	−26.39	<0.0001

*R = 0.32, R^2^ = 0.10, AdjR^2^ = 0.10*.

To further determine the factors influencing anxiety symptoms among Chinese adolescents, we conducted a multiple linear regression analysis, and the model was highly significant (*R* = 0.25, *R*^2^ = 0.06, Adj*R*^2^ = 0.05, *p* < 0.001) (Table 4). Multivariate analyses revealed that older age, female sex, living in urban areas, living in severe epidemic areas, symptoms related to COVID-19 during the past 14 days, and conflicts with parents during home quarantine were identified as risk factors for anxiety. Among these risk factors, symptoms related to COVID-19 had the strongest impact on anxiety. Meanwhile, focusing on epidemic information (<3 h/day), communication with peers, exercising, and receiving public welfare psychological assistance during the pandemic were found to be protective factors for anxiety symptoms.

**Table 4:** Results of multiple linear regression analysis of influencing factors for anxiety symptoms.

**Variables**	**B**	**SE**	**ß**	***T***	***p***
(Constant)	6.08	0.12		50.23	<0.0001
Gender	0.69	0.02	0.10	41.15	<0.0001
Age (years)	0.25	0.02	0.04	14.83	<0.0001
Residential areas	0.04	0.02	0.01	2.56	0.01
Situation of COVID-19 epidemic in residence	0.27	0.02	0.03	14.57	<0.0001
Having symptoms related to COVID-19	3.11	0.11	0.07	28.93	<0.0001
Family economic situation	0.01	0.02	0.01	0.23	0.82
Conflict with parents during home quarantine	1.26	0.02	0.12	49.76	<0.0001
The attention to epidemic information per day	−0.93	0.04	−0.06	−24.79	<0.0001
Communication with peers	−0.66	0.01	−0.12	−48.98	<0.0001
Exercise during the epidemic	−0.32	0.01	−0.04	−18.60	<0.0001
Receive public welfare psychological assistance during the epidemic	−0.25	0.01	−0.03	−14.39	<0.0001

*R = 0.25, R^2^ = 0.06, AdjR^2^ = 0.05*.

Next, we performed a multiple linear regression analysis to identify factors associated with PTG. As indicated in Table 5, the model was highly significant (*R* = 0.35, *R*^2^ = 0.12, Adj*R*^2^ = 0.12, *p* < 0.001). The results identified that sex, residential areas, COVID-19 epidemic in residence, symptoms related to COVID-19, family economic situation, conflict with parents during home quarantine, attention to epidemic information per day, communication with peers, exercising, and receiving public welfare psychological assistance during the epidemic were significant predictors of PTG in adolescents. Communication with peers played the most important role in PTG.

**Table 5:** Results of multiple linear regression analysis of influencing factors for PTG.

**Variables**	**B**	**SE**	**ß**	***T***	***p***
(Constant)	16.7	0.69		24.01	<0.0001
Gender	−3.84	0.09	−0.09	−39.6	<0.0001
Age (years)	0.20	0.09	0.01	2.03	0.04
Residential areas	−0.75	0.09	−0.02	−7.59	<0.0001
Situation of COVID-19 epidemic in residence	−1.37	0.11	−0.03	−12.82	<0.0001
Having symptoms related to COVID-19	−2.02	0.62	−0.01	−3.27	0.001
Family economic situation	1.12	0.13	0.02	8.76	<0.0001
Conflict with parents during home quarantine	−4.55	0.15	−0.07	−31.32	<0.0001
The attention to epidemic information per day	6.36	0.21	0.07	29.72	<0.0001
Communication with peers	8.06	0.08	0.24	104.15	<0.0001
Exercise during the epidemic	7.06	0.09	0.16	71.26	<0.0001
Receive public welfare psychological assistance during the epidemic	1.83	0.09	0.04	18.65	<0.0001

*R = 0.35, R^2^ = 0.12, AdjR^2^ = 0.12*.

## Discussion

Our study was conducted during the rapid rise period of the COVID-19 outbreak (11), which covered 31 provinces, centrally administered municipalities, and autonomous regions in mainland China. This survey indicated that adolescents had a significantly higher prevalence of depression (35.9%) and anxiety (28.0%) during the COVID-19 outbreak compared with the national data in the normal period, which showed a lower incidence of depression (3.6%) and anxiety (4.3%) in adolescents (12). In other words, exposure to the pandemic had a psychological impact on adolescents, and close attention and great efforts were required to effectively address these emergency issues. Notably, 45.6% of adolescents experienced PTG. These results were consistent with our hypothesis that the pandemic offered both challenges and opportunities for adolescents. Furthermore, the current study explored the factors influencing the reduction of negative emotions and promotion of the capacity of PTG during public health emergencies.

### The Influence Factors of Negative Emotions in Chinese Adolescents During the COVID-19 Pandemic

Adolescence is a key period for the development and improvement of cognition, emotion, and personality. During this critical stage, the depression and anxiety caused by such a stressful event would inevitably affect adolescents' physical health, social interaction, and academic achievement (13). From a personal perspective, our results found that sex, age, severity of the pandemic in the place of residence, and symptoms related to COVID-19 were associated with anxiety and depression. Female adolescents had increased anxiety and depression compared with males, and an association between female sex and psychological distress has been consistently found in previous studies (14). In addition to sex, adolescents aged 21–26 years old were found to have higher levels of depression and anxiety than adolescents aged 16–20 years old, which was consistent with a recent study indicating that adolescents with higher grades had a greater prevalence of depressive and anxiety symptoms (10).

Surprisingly, about 30% of adolescents in slight epidemic areas suffered depression and anxiety, which was similar in severely epidemic areas. This phenomenon can be explained by the emotional resonance effect, whereby the pandemic as a sudden stressful event caused adolescents' stress, anxiety, and depression to eventually transmit to others, regardless of the severity of the epidemic in their residential area. However, our results showed that the level of negative emotions in severe epidemic areas was higher than that in slight epidemic areas, which indicates that the degree of anxiety and depression is much more serious in severe epidemic areas. In addition, adolescents with symptoms related to COVID-19, such as fever and cough, felt fear and helplessness and were extremely worried about catching COVID-19. Especially in a state of isolation at home, the shortage of medical supplies and resources, or even quarantine, could aggravate their negative emotions (2). Meanwhile, our findings illustrated that if adolescents pay extreme attention to epidemic information, regardless of the positive or negative information that exceeds the bottom line that their psychology can tolerate, it will naturally trigger vicarious trauma and cause serious mental problems (15). Therefore, it is crucial for adolescents to reasonably organize time and maintain an appropriate state of concern.

From the family's perspective, our findings demonstrated that conflict with parents during home quarantine was a risk factor for depressive and anxiety symptoms. Most Chinese adolescents aged 16–26 years old are from single-child families, and their parents are typically characterized by high authority, which can cause parent–adolescent conflicts (16). Our investigation revealed that the epidemic made adolescents spend more than 10 h per day with their parents, lasting nearly 2 months, which led to escalated conflicts in 14.1% of Chinese families in our study. According to the McMaster model of family functioning, family function can provide a healthy environment for adolescents in physical, psychological, and social development (17). Thus, parents are advised to focus on adolescents' personal choices and maintain good interactions with them to reduce the impact of long-term home isolation.

From a social perspective, regular online communication with peers is a protective factor in reducing the levels of anxiety and depression. Peers played the role of social support in adolescent development. According to the stress, social support, and buffering hypothesis, social support can relieve pressure and maintain an individual's physical and mental health under stress conditions (18). Therefore, online peer-to-peer communication should be encouraged to provide social support and buffer against the negative effects of home confinement.

From a national perspective, guidance on recommended health behaviors through various media needs to be delivered to the public, such as home exercises including gymnastics, yoga, and Tai Chi. The results of our study showed that active engagement in physical exercise can reduce anxiety and depression. Meanwhile, our results indicated that psychological counseling hotlines or online public welfare services should be provided to alleviate the emotional problems caused by the epidemic, especially for adolescents (19).

### The Influence Factors of PTG in Chinese Adolescents During the COVID-19 Pandemic

During the pandemic, we investigated the activated first-level emergency response in all provinces and autonomous regions (20). Under such circumstances, although negative emotions were found in adolescents in our study, nearly half of them were not knocked down by the epidemic, which in turn activated their anti-frailty ability (21). Among these positive reactions, “spiritual growth” was one of the most prominent themes that emerged in adolescents, such as finding the meaning of life and appreciating their health, family, and friends. These positive cognitions, beliefs, and emotional changes were gained due to their attempts to cope with the pandemic (22), which established PTG in adolescents during the public health emergency (23).

Our results showed that men had higher levels of PTG than women. PTG theorists [e.g., (24)] suggested that gender differences in reported PTG are more vulnerable to emerge around puberty, which could further clarify the current mixed results (25). The reason for this may be that threat appraisals and family support partially mediated the relationship between sex and growth, as males experienced a lesser sense of danger and lesser family support than females, thus eventually increasing PTG (26).

Multiple linear regression analysis suggested that adolescents' family economic status is positively correlated with PTG, which was similar to a previous research indicating that a better family economic situation predicted a higher level of PTG (27). It is particularly important that PTG was greatly influenced by variables, including the relationship with parents and peers, exercising according to the guidance, and receiving online psychological assistance. During the pandemic, a harmonious family atmosphere can help adolescents change their irrational cognitive beliefs and have greater hope and optimism about the future. This was consistent with a recent study that revealed a higher level of warmth in parenting to be a possible mechanism facilitating PTG in the child's response to traumatic events (25). Furthermore, according to the PTG theoretical model (6), a “good peer relationship” was proposed for trauma intervention. Peer support providing a new cognitive schema or a new perspective on trauma may be conducive to reducing feelings of isolation (28). Our findings also demonstrated that health behavior guidance, psychological assistance, and crisis intervention can serve as valuable resources to help adolescents achieve PTG.

### The Advantages and Limitations of This Study

As the COVID-19 epidemic continues to spread, our findings provide theoretical guidance for psychological support creation for countries and regions. Our findings indicate that equal attention should be given to adolescents to maintain their mental health in both slight and severe epidemic areas. We also suggest that adolescents should frequently communicate with peers online, enhance their interaction with parents, and engage in sustainable exercise to relieve the psychosocial stress caused by home confinement. When faced with psychological problems that cannot be resolved, adolescents should turn to the public welfare psychological platform to seek assistance. Parents need to pay more attention to adolescents' needs, respect their identity instead of controlling them, and try to create a harmonious family atmosphere. Furthermore, it is essential to establish an official online platform to provide information and mental health consultation for the public. Finally, the efforts of individuals, families, and society are highly recommended to win the confidence of adolescents and turn challenges into opportunities.

This study had some potential limitations. Firstly, this is a cross-sectional study, which means that we cannot infer causal relationships between the variables; additional longitudinal studies should be conducted in the future. Secondly, another limitation is the self-report measures, which may have a response bias when assessing the psychosocial variables. Thirdly, there are various psychological effects of the COVID-19 pandemic; however, this study mainly focused on the aspects of anxiety, depression, and PTG. Furthermore, a more comprehensive research (e.g., suicide risk) on the COVID-19 pandemic should be carried out and relevant management plans should be provided as well. Finally, although the survey conducted online was suitable for rapid assessment, some response bias may have affected the results.

## Conclusion

During the rapid rise period of the COVID-19 outbreak, we identified that 35.9% of adolescents experienced depression and 28.0% experienced anxiety. Surprisingly, about 30% of adolescents in a slight epidemic area reported anxiety and depression, which was almost similar to that in severe epidemic areas. However, the level of negative emotions was higher in severe epidemic areas. Our study also found that nearly half of the adolescents experienced PTG, which confirmed that challenges and opportunities coexist for adolescents faced with the pandemic. Female sex and older age were associated with higher levels of depression and anxiety and lower levels of PTG. Living in urban areas, symptoms related to COVID-19, excessive attention to epidemic information, and conflict with parents during home quarantine were independent risk factors for depression, anxiety, and PTG. Peer communication, exercising, and receiving public welfare psychological assistance were protective factors. Poor family economic status was a significant risk factor for depression and PTG. Our findings contribute to identifying high-risk groups in adolescents and provide a foundation for formulating psychological interventions to improve adolescents' mental health. This study demonstrated that adolescents, families, and society should make joint efforts to win the current war against COVID-19.

## Data Availability Statement

The original contributions presented in the study are included in the article/supplementary materials, further inquiries can be directed to the corresponding author/s.

## Ethics Statement

The studies involving human participants were reviewed and approved by Ethics Committee of Harbin Medical University (HMUIRB20200002). Written informed consent to participate in this study was provided by the participants' legal guardian/next of kin.

## Author Contributions

JZ, YY, ZQ, and XY: conceived and designed the experiments. JH, JL, XL, and YZ: performed the experiments. TB and HC: data analyses. ZQ and SZ: contributed reagents, materials, and analysis tools. JZ and YW: wrote the manuscript. All authors have reviewed the manuscript and contributed to the manuscript and approved the submitted version.

## Funding

This research was supported by the National Natural Science Foundation of China (81773536 to YY) and the China Postdoctoral Science Foundation (2019M651311 to JZ).

## Conflict of Interest

The authors declare that the research was conducted in the absence of any commercial or financial relationships that could be construed as a potential conflict of interest.

## Publisher's Note

All claims expressed in this article are solely those of the authors and do not necessarily represent those of their affiliated organizations, or those of the publisher, the editors and the reviewers. Any product that may be evaluated in this article, or claim that may be made by its manufacturer, is not guaranteed or endorsed by the publisher.
